# Glyphosate, Roundup and the Failures of Regulatory Assessment

**DOI:** 10.3390/toxics10060321

**Published:** 2022-06-13

**Authors:** Eva Novotny

**Affiliations:** Clare Hall, University of Cambridge, Herschel Road, Cambridge CB3 9AL, Cambridgeshire, UK; en@en.eclipse.co.uk

**Keywords:** glyphosate, Roundup, regulation, assessment, EFSA, ECHA, EPA

## Abstract

Roundup is the most widely used herbicide in agriculture. It contains glyphosate as the ‘active ingredient’, together with formulants. There are various versions of Roundup, with somewhat different effects depending on the formulants. Most genetically-modified crops are designed to tolerate Roundup, thus allowing spraying against weeds during the growing season of the crop without destroying it. Having been so heavily used, this herbicide is now found in the soil, water, air, and even in humans worldwide. Roundup may also remain as a residue on edible crops. Many studies have found harm to the environment and to health, making it imperative to regulate the use of Roundup and to ensure that its various formulations pose no danger when used in the long-term. Unfortunately, regulators may only assess the ‘active ingredient’, glyphosate, and ignore the toxicity of the formulants, which can be far more toxic than the active ingredient. This omission is in violation of a ruling by the Court of Justice of the European Union. There are close ties between the regulators and the industry they are supposed to regulate. Objectionable practices include ‘revolving doors’ between the regulators and the industry, heavy reliance on unpublished papers produced by the industry while dismissing papers published by independent scientists, and strong covert influence on the regulatory process by industry. Although this paper focuses on the European Union (EU), the situation is much the same in the United States.

## 1. Introduction

Since the first appearance of genetically modified (GM) crops on the market in the mid-1990s, the herbicide Roundup that they were designed to tolerate has been increasingly in use. Roundup is now the most widely used herbicide in the world. Most GM crops are engineered to tolerate it including some that were primarily designed for other traits. Much damage has been caused to the environment such as the emergence of ‘superweeds’, the contamination of waterways, and a decline in soil fertility. Farm animal health has also suffered from glyphosate and Roundup. Torretta et al. [1] provided a review covering many topics regarding glyphosate and its products including its chemical properties; the debate over their safety among the developer (Monsanto Company, St, Louis, USA) and the European Food Safety Authority, the Food and Agricultural Organisation, the World Health Organisation agencies charged with evaluating the safety, and others; the contamination of soil, water, air, food and some fibres in daily use; studies on the effects on human and animal health; and alternatives to the use of glyphosate. There can be little doubt that humans are negatively affected, although ethical constraints prevent research on humans to confirm this. Claims have been made by the genetic modification (GM) industry that millions of meals of GM crops (most of them sprayed with Roundup) have been eaten by people with no ill effect; but the claim has no scientific basis. In fact, Swanson et al. [2] showed that strong correlations existed between the increasing use of Roundup and the increasing rise in the number of Americans suffering from one or more of the 22 chronic illnesses in the study, which included obesity, hypertension, senile dementia, and several types of cancer.

With so much potential to cause harm, Roundup should be carefully assessed and monitored and needs to be distinguished from its declared active ingredient, glyphosate. Roundup formulations generally contain about 40% glyphosate together with adjuvants, which increase its effectiveness, and other co-formulants, according to Séralini and Jungers [3]. Some of these ingredients are toxic, according to Hao et al. [4]. The ingredients of any Roundup formulation are commercially confidential and are not available to the public, but one commonly known adjuvant is the toxic surfactant polyoxyethylene amine (POEA).

Although the Court of Justice of the European Union [5] has ruled that the authorisation procedure must include the assessment not only of the active substances but also of their cumulative effects and their effects when combined with the other substances in the product, this is not always conducted.

In light of the debate over the safety of glyphosate, there are now versions of Roundup that do not contain the declared active principle; glyphosate has been replaced by substances that are supposedly less toxic such as pelargonic acid. These formulations, however, are also toxic, as found by Séralini and Jungers [3]. The new formulants may include heavy metals and polycyclic aromatic hydrocarbons at levels that may be hundreds or thousands of times the levels in water. This inclusion of undeclared toxic chemicals violates the EU rules on pesticides.

In both the European Union (EU) and the United States, regulatory agencies have been infiltrated by the pesticide and GM industries and therefore continue to maintain that glyphosate, and therefore Roundup, is a safe herbicide. The previous assessment in the EU, in 2017, allowed for glyphosate to be used until 15 December 2022. A new assessment is underway and was meant to be completed by that date. However, the authorities have received so many responses to consultations that they cannot consider them all in time to meet the deadline. On 10 May 2022, the EFSA announced that EFSA’s peer review is planned to be ready in July 2023 [6].

At the time of writing this paper, it appears that on glyphosate, and hence Roundup, a verdict of safety is again likely [7,8].

Székás and Darvas [9] discussed the various pertinent subjects of the history of the risk assessment of pesticides, the numerous studies on glyphosate and its co-formulants showing harm to health, the contamination of waterways, residues in food, and the debates over safety between various agencies. Robinson et al. [10] described how the assessment process in the EU is governed by ‘hard’ and ‘soft’ laws, which are not fully implemented and that may not even suit the purpose; how scientific misconduct plays an important role in assessments; how regulators dismiss most peer-reviewed studies in favour of industry-sponsored studies; how scientific methods may be used in a flawed way; how regulation suffers from a lack of independence of the regulatory agencies; and what improvements should be made to ensure the safety of the approved products.

## 2. How the EU Assesses the Safety of Glyphosate

### 2.1. The Major Players

The plan being followed to assess the safety of glyphosate has been set out by the European Food Safety Authority (EFSA) [11,12]. The bodies prominently involved in the EU assessment and approval process are as follows.

The applicant, or group of applicants, is sometimes referred to as the Glyphosate Renewal Group (GRG). In the current assessment, the GRG comprises eight companies including Bayer/Monsanto, who are involved in the manufacture or sale of glyphosate, in addition to other interested parties [13].

The European Commission appoints an expert group from the EU Member States, which serve as Rapporteurs (RMS). This group is also sometimes referred to as the Assessment Group on Glyphosate (AGG). In the current assessment, these countries are France, Hungary, the Netherlands, and Sweden. At the conclusion of the assessment phase, the Commission is involved in the approval phase.

The European Food Safety Authority (EFSA) provides an assessment of the risk to humans, animals, and the environment due to exposure to glyphosate [14].

The European Chemicals Agency (ECHA) assesses the hazard of glyphosate and classifies it on the basis of carcinogenicity, genotoxicity, mutagenicity, reproductive toxicity, and specific organ toxicity [14,15].

Member States participate through their competent authorities in the assessment, and vote in the approval stage.

### 2.2. The Assessment Procedure

A skeletal outline of the whole assessment and approval procedure now follows. Although it is written in the present tense, some events are from the past and some are still in the future at the time of writing.

To begin the renewal process, the group of applicants must submit an application and a comprehensive dossier to the Rapporteur Member States. Their assessment is based on studies provided by the group of applicants as well as studies in the public literature. Their report is passed onto the EFSA and ECHA, which assess the information and publish their draft reports on their websites. Public consultations are then held (which closed on 22 November 2021). Comments from the consultation are then addressed by the Rapporteur Member States, and their report is sent to the ECHA for its classification of glyphosate (due in May/June 2022). Additional information can then be requested from the applicant group, after which the Rapporteur Member States prepare an updated renewal draft to the EFSA. A peer review is then conducted by the EFSA with the collaboration of the Member States [14]. It is then the task of the EFSA to correlate all the information and to pass its conclusion to the European Commission, which also receives the opinion of the ECHA. The EU Commission then prepares a draft renewal report that is sent to all Member States. At this stage, the applicants are consulted on the draft renewal report. Following discussions between the European Commission and Member States, a vote is undertaken by the Member States on the European Commission’s proposal to decide whether glyphosate may be approved for use within the EU. Once approved, individual Member States must take the responsibility to assess the safety of any product containing glyphosate that will be used in that country [13,14]. However, the Court of Justice of the European Union has stated that it is the responsibility of the applicant to prove that a pesticide (or, more generally, ‘plant protection product’) causes no immediate or long-term harm to human health such as carcinogenicity or toxicity [5]. The Court concluded that it is the responsibility of the competent authorities of the Member States to verify the applicant’s assurances of safety. Approval of an active substance must be reviewed within a limited time [8].

## 3. Regulatory Faults

### 3.1. General Comments

In 2004, a former British Minister of State for the Environment, Michael Meacher, wrote in a Foreword to a book on GM crops [16] that it “tells us a great deal about how power is exercised today—funding political parties and key individuals, networking around opinion-formers and decision-makers and fixing strategic job swaps between the biotech industry and Government” [i.e., ‘revolving doors’]. Little has changed since then. In the European Union and in the United States, regulations are designed more to further the industry they are meant to regulate rather than to protect the environment and the health of the populace.

Four major issues corrupt the regulatory process: (1) failure to distinguish between glyphosate and Roundup; (2) ‘revolving doors’ between a regulatory authority and the industry it is meant to regulate; (3) reliance for safety data on unpublished industry documents while largely ignoring publications by independent scientists; and (4) covert influence by the industry.

### 3.2. Failure to Distinguish between Glyphosate and Roundup

The EU-wide regulatory approval process only tests the designated active ingredient in a pesticide formulation, not the formulation that is actually in use. This is an important distinction because the formulants in Roundup herbicide are far more toxic than the active ingredient, glyphosate, by factors of about 1000 (Séralini et al. [17]). Moreover, the formulants are also endocrine disrupters and nervous system disruptors.

Defarge et al. [18] found that formulants, not glyphosate, were the main causes of the toxic and endocrine-disruptive effects of the 14 glyphosate-based pesticides they studied.

The toxicity of nine pesticide formulations including one based on glyphosate was studied by Mesnage et al. [19]. Roundup proved to be 125 times more toxic than glyphosate. This result should be of concern to regulators, as it casts doubt on the relevance of the concept of the acceptable daily intake (ADI) for Roundup. The ADI is based on tests that feed animals various foods containing the active principal of a herbicide, but not the whole formulation. The study concludes that the present ADI, which is 0.3 ppm, should perhaps be 3 ppb.

### 3.3. Revolving Doors at the EFSA

A major player in the assessment of new food and feed products is the EFSA. Unfortunately, this body has a history of acting more to protect the GM and chemical industries than to ensure the safety of the food consumed by the public. Over years, it has been asked repeatedly by the European Parliament to reform its working practices, especially its close ties to industry. A report in 2012 described its malpractices, and the European Parliament postponed the discharge of the EFSA’s budget for six months because of its bad management of conflicts of interest [20,21]. In June 2017, the EFSA adopted a document entitled “EFSA’s policy on independence: How the European Food Safety Authority assures the impartiality of professionals contributing to its operations” [22]; but in the same month, it was found that nearly half the experts at the EFSA had conflicts of interest [20,21,23]. A non-exhaustive chronology of events at the EFSA showed that problems still remained a few years later [21]. A new policy for reforming the Authority was issued in March 2021: “Transparency in risk assessment: a new era begins”, which promised that “New rules on transparency and sustainability are set to transform the way EFSA carries out its role as risk assessor in the EU food safety system” [24].

The European Chemicals Agency (ECHA), which has responsibility for assessing the safety of chemicals, has also been challenged by Greenpeace [25] over its policy on conflicts of interest. When it was due to publish its assessment of glyphosate in 2017, the chairman and two members of the Risk Assessment Committee had conflicts of interest that violated the Agency’s own rules.

### 3.4. Reliance on Unpublished Industry Documents

In addition to conflicts of interest, the EFSA has also been guilty of relying heavily on unpublished company documents to guide its assessments. In an exchange of letters between the ECHA and Greenpeace, the ECHA replied that “The EU legislation requires us to work with unpublished studies undertaken or commissioned by industry” [26].

The four Rapporteur Member States, in their report to EFSA and ECHA, stated that they “have examined all the evidence submitted by the companies that are seeking renewed approval to market the substance in the EU” [11]. This document does not mention any other sources of evidence.

Criticisms have been made about the heavy dependence on unpublished industry-sponsored studies (which find that glyphosate is safe) and the dismissal of studies by independent scientists (which almost invariably find that it is harmful). This inequality in assessment arises from the EU requirement that “All studies must be carried out according to Good Laboratory Practice (GLP) in approved laboratories. These laboratories are under supervision of national authorities” [8,27,28]. GLP, however, does not set out rules for excellence in the conduct of scientific studies; rather, GLP was intended to ensure greater reliability and accountability with strict documentation and further controls in studies supplied to regulatory bodies. Nevertheless, some of the studies that were compliant with GLP and were used in the 2017 approval of glyphosate proved to be based on fraudulent evidence [10,27,28]. The consequence of the requirement of compliance with GLP is that many published studies are ruled out of consideration in the assessment of glyphosate. Adherence to GLP does not guarantee the reliability of a study, nor does non-adherence signal unreliability. Unlike most industry-sponsored studies, those carried out by independent scientists are normally peer-reviewed and published, thus allowing the rest of the scientific community to examine them and find any faults. A critique “notes that more than 90% of the scientific literature on glyphosate had been ruled unreliable or irrelevant by the regulators, leaving the assessment of risk based on data provided by industry” [29].

The chemical expert, Tony Tweedale, speaking to GM Watch [30], analysed the EFSA’s updated literature review, which concluded that glyphosate was not an endocrine disruptor. The review had been conducted by a consultancy firm on behalf of the Glyphosate Task Force, a coalition of pesticide companies led by Monsanto Company. Tweedale said that there were 104 published studies on the subject, of which a significant number concluded that glyphosate was an endocrine disruptor; but every one of them had been rejected. Seven studies did not meet the criteria for ‘relevance’ and ‘reliability’ as defined by three employees of the chemical giant BASF, but “97 (93%) were excluded just by screening the title and/or abstract”.

In 2015, the international Agency for Research on Cancer (IARC), an expert body under the World Health Organisation, published its adverse assessment on glyphosate and glyphosate-based products (see Section 5.2) [31]. This condemnatory report was fiercely opposed by proponents of pesticides, who contested the IARC’s choice of limiting their evidence to published, peer-reviewed studies and publicly available government reports [32]. In contrast, the regulators primarily used publicly unavailable, unpublished studies supplied by the industry.

### 3.5. Covert Influence of Industry

During the assessment of glyphosate completed in 2017, the EFSA asked its Rapporteur Member State, Germany, for an updated review of the scientific literature. This was performed by the German Federal Institute for Risk Assessment (BfR). However, the BfR did not conduct its own review (because they found the number of industry papers overwhelming) but supplied a review taken from the work of the industry’s Glyphosate Task Force, which was amended for the purpose [33,34].

Furthermore, during the glyphosate evaluation of 2017, email correspondence between the EFSA and industry lobbyists revealed that the EFSA had, according to its normal process, allowed industry representatives to have advanced access to its final evaluation in 2015 [35]. The representatives were asked about what should be redacted and it appears that they made changes to the conclusions and background documents. The EFSA said that these were normally limited to “factual errors or typos”. NGOs were forbidden advanced access.

Glyphosate was declared to be non-carcinogenic by the EFSA, ECHA, and BfR by violating the rules that guide such assessments [36]. According to the rules, a substance is to be considered carcinogenic if two independent studies on animals find an increased incidence of tumours; but the three authorities above dismissed the seven studies showing an increase in tumour incidence out of the twelve such studies.

Four Members of the European Parliament and others requested access to unpublished studies about the toxicity and carcinogenicity of glyphosate, but the EFSA refused in order to protect the company’s commercial interests. However, the EU General Court overturned the EFSA’s decisions [37].

When IARC was working on its upcoming 2015 report on glyphosate [31,32], Monsanto feared the outcome and plotted to cast doubt on the report. The published report did indeed find glyphosate harmful, and Monsanto’s plans to counteract this alarming verdict were exposed by the Monsanto Papers, as explained by Kehr et al. [38] and will now be summarised. An email from the man leading Monsanto’s Regulatory Product Safety Assessment stated that they would use a strategy employed previously, namely, ‘ghostwriting’ some articles under the names of academic toxicologists who would “just edit and sign their names”. An earlier ghostwritten paper that had been published under the names of three ‘independent’ scientists was entitled *Safety evaluation and risk assessment of the herbicide Roundup and its active ingredient, glyphosate, for humans* [39]. This paper found no fault with glyphosate and was widely quoted including to regulatory agencies in support of the safety of glyphosate and Roundup. It had been written to counteract the report of a toxicologist, James Parry, who had been contracted by Monsanto and found that the glyphosate formulations showed evidence of carcinogenic effects. The ghostwritten paper succeeded in is intent and came to replace the one by the toxicologist.

The Monsanto Papers also revealed how the company attempted to destroy the credibility and reputation of the lead author, G.-E. Séralini, of a paper finding adverse health effects in rats fed Roundup or a GM maize or both [40]. Although the paper had already been published following a favourable peer review, it was massively attacked on fabricated grounds [41,42]. The paper was then retracted for being “inconclusive”, a reason not recognised in scientific publishing. However, the paper was later re-published in another journal [43] (see also Section 5.2).

## 4. Regulation in the United States

The regulatory agencies in the United States that are responsible for the safety of food are the Environmental Protection Agency (EPA) and the Food and Drug Administration (FDA). The EPA is responsible for setting the tolerance levels of pesticides to ensure safety to health and the environment [44]. The FDA is charged with ensuring that the amount of pesticide residues on food and animal feed is within the tolerances set by the EPA [45].

Many of the same failures of regulation occur in the United States as in the European Union. In her book *Whitewash: The story of a Weed Killer, Cancer, and the Corruption of Science*, Gillam [46] (p. 114) wrote about the corrupt influence of Monsanto Company and others in the United States: “What the records clearly show is a roster of U.S. academics—people employed by taxpayer-funded institutions—quietly working with Monsanto, other agrochemical companies, and public relations experts to tout the benefits of company products, to counter anything that points to problems with glyphosate or glyphosate-tolerant crops, and to cripple unfavourable legislation or regulation”.

Evidently, neither the EPA nor the FDA is doing its job properly, as testing of 83 foods by The Detox Project [47] showed that 45 had high levels of glyphosate residues, the maximum being 1150 ppb in one brand of 100% whole wheat bread.

The high levels of glyphosate found in food reflects the EPA’s lack of concern about the herbicide: “The EPA continues to find that there are no risks of concern to human health when glyphosate is used in accordance with its current label. EPA also found that glyphosate is unlikely to be a human carcinogen” [44].

## 5. Consequences for Environment and Health

The formulation of Roundup has effects on the environment and health that differ from those of its ‘active ingredient’, glyphosate. Both are harmful but in different ways.

### 5.1. Effects of Roundup and Glyphosate on the Environment

The Soil Association has provided an overview of the effects of glyphosate on soil and soil organisms [48], finding that some topics evoke opposing views in the literature. The overview describes the following effects: the sorption of glyphosate onto soil and the risk of leaching; degradation of glyphosate; the effect of glyphosate on soil micro-organisms; the effects on soil microbial community population, function, and structure; the impact on mycorrhizal fungi; impact of repeated glyphosate applications; increase in micro-organisms causing disease in crops; and the impact on soil fauna and earthworms.

In 1996, Monsanto marketed the first ‘Roundup Ready’ crops, which were genetically engineered to tolerate the application of Roundup. The use of Roundup then began to rise markedly, especially on Roundup Ready soya beans, cotton, and maize (corn), which were widely planted. Thirteen years later, Benbrook [49] reported that repeated use of Roundup on the same land resulted in the emergence of weeds that were resistant to Roundup. A particularly noxious ‘superweed’ is Palmer amaranth, which first became resistant to Roundup in one place in the United States, but quickly spread from infesting 500 acres to about 1,000,000 acres in four years. It is an especially difficult weed to control because it grows very rapidly and is so strong that it can damage harvesting equipment. Some farmlands have been abandoned because the weed could not be controlled. Over time, the number of Roundup-resistant weeds has been increasing, and older pesticides have come back in use.

A review by Van Bruggen et al. [50] described many instances of the adverse effects of glyphosate on the environment and on health (see Section 5.2 for the effects on health). It must be noted that the authors did not distinguish between glyphosate and glyphosate-based herbicides, and this lack of distinction therefore applies to the examples below. Among the effects on the environment are the following:

∗ Glyphosate and its degradate aminomethylphosphonic acid (AMPA) accumulate in the soil and may affect water. These may persist in soils with a high clay content for more than a year.
∗ Glyphosate has also been detected in the air and rain at certain seasons. Runoff from urban areas flows into streams and rivers, and ultimately flows into seawater, where it is highly persistent.
∗ Glyphosate and AMPA decrease photosynthesis.
∗ Glyphosate and AMPA are commonly found in drinking water at levels below the accepted daily intake established in 1997.
∗ Residues of glyphosate and AMPA are found in plant products.
∗ Plants subjected to glyphosate do not produce certain compounds that protect them against root pathogens, and they may die from infection.
∗ Glyphosate applied at recommended doses or lower have a negative effect on micro-organisms that promote plant growth.

A study by Santadino et al. [51] of the effects of glyphosate on earthworms found that while the population of controls increased in time, populations treated with either the regular dose for perennial weeds or double that dose decreased. This raises the possibility that earthworms subjected to glyphosate might become extinct, with consequences for soil fertility.

The effects of the commercial formulation Roundup^®^ (R450) on the soil filamentous fungus *Aspergillus nidulans* were investigated by Nicolas et al. [52] The median lethal dose applied was a dilution of 1% of that used in agriculture. The formulation was far more active than technical glyphosate. Growth, cellular polarity, endocytosis, and mitochondria were adversely affected at the median lethal dose and lower. The results suggest that soil ecosystems could be impaired.

Hebert et al. [53] found that phosphorus is released into the soil from the glyphosate molecule as it degrades. Although the amount of phosphorus that has accumulated in soil over the years of glyphosate use on crops is still quite low, it has reached a significant level. Phosphorus also leaches from the soil into agricultural waterways, which can suffer from nutrient pollution.

### 5.2. Effects of Roundup and Glyphosate on Health

Between 1978 and 1986, Monsanto conducted experiments on rats, mice, and dogs to test glyphosate for toxicity [54]. These were submitted to the U.S. Environmental Protection Agency but were treated as being commercially confidential and were not available in the public domain. However, the EPA did release some Memos from the early 1980s showing significant damage to the kidneys of rats, together with other changes that could be an early step in tumour formation. Further tests on mice in 1983 found numerous adverse changes in organs. An EPA Memo in 1991 revealed that EPA experts knew before 1985 that glyphosate caused pancreatic, thyroid, and kidney tumours. Despite this evidence and many more recent studies, the EPA continues to maintain that glyphosate is not a concern for human health when used in accordance with the instructions on the label, and that it is also unlikely to be carcinogenic to humans [44].

The effects of a genetically modified (GM) maize and of Roundup on rats were investigated in a two-year study by Séralini et al. [40,43]. Adverse health effects were found including tumour formation and early deaths. The number of non-regressive palpable tumours was two to three times higher in the treated female rats than in the controls. Three tumours developed in the treated male rats, and one tumour developed among the control males. Mortality was earlier and two to three times higher among the treated females than in the controls, but the results were mixed for males. Critics of the study who insisted that Roundup (and GM crops) was safe claimed that too few rats (10 per group) had been used in this carcinogenicity study, as they erroneously called it. It was, in fact, a toxicological study; therefore 10 rats per group sufficed. In carcinogenicity studies, 60 rats per group are required to lower the risk of not finding a rare event. The fact that so many ‘rare’ events of tumour formation occurred with so few animals actually strengthens the conclusion that Roundup causes harm to health.

The IARC evaluation of glyphosate and of glyphosate-based herbicides containing carcinogenic formulants [31] concluded that “There is *limited evidence* in humans for the carcinogenicity of glyphosate. A positive association has been observed for non-Hodgkin lymphoma” and “There is *sufficient evidence* in experimental animals for the carcinogenicity of glyphosate”. The report also found strong evidence of DNA damage in a variety of animals and in in vitro studies on human tissues. There is also strong evidence that glyphosate, glyphosate-based formulations, and the major metabolite of glyphosate, aminomethylphosphonic acid (AMPA,) induces oxidative stress in animals and in human cells in vitro. In addition, evidence was found for chromosomal damage in humans exposed to a glyphosate-based herbicide.

Defarge et al. [18] investigated the health effects of glyphosate and 14 of its pesticide formulations on three human cell lines: HepG2, HEK293, and JEG3. They found that the formulants, and not glyphosate, were the main causes of the toxic and endocrine-disruptive effects. In addition, 11 of the formulations contained the heavy metals arsenic, chromium, cobalt, lead, and nickel, which are also toxic and endocrine-disrupting.

The review by Van Bruggen et al. [50] describes the following effects of glyphosate and AMPA:

∗ Male rats had decreased fertility when given a low dose of glyphosate over an extended time, but there was no such effect when given a single high dose. Cell cultures of cattle ovaries similarly showed decreased function at low dose, but no effect at high dose. This correlation between dose and effect is typical of endocrine-disrupting substances.
∗ Glyphosate and the surfactants polyoxyethylene amine (POEA) and MON 0818 (which contains POEA) have negative effects on aquatic animals in the food web.
∗ Glyphosate affects the interaction between fish and their pathogens and parasites.
∗ Animal feed or water contaminated with glyphosate can negatively affect intestinal bacteria, leading to reduced animal health. Intestinal fungi are also affected.
∗ There are observed associations between glyphosate resistance and antibiotic resistance in bacteria. These resistant bacteria could be transmitted from agricultural fields to animals and humans.
∗ Glyphosate may be a driver for antibiotic resistance.

A study within the Puerto Rican PROTECT project by Silver et al. [55] of pregnant women found that pre-term births were significantly associated with higher levels of glyphosate in their urine when measured around week 26 of the pregnancy. However, this correlation did not occur in the early weeks of pregnancy. Similar results were obtained for AMPA.

In Argentina, Avila-Vazquez et al. [56] studied a small agricultural settlement exposed to the environmental use of a glyphosate-based herbicide containing carcinogenic formulants. The glyphosate was initially concentrated and then prepared for application in the settlement. The investigators examined the water, soil, and particulate-matter contamination, and also conducted an epidemiological appraisal of cases of cancer, abortion, and genetic abnormality. Spontaneous abortion occurred three times more often than in the national population, and genetic abnormality occurred twice as often. Cancer rates were two to three times higher in terms of incidence, prevalence, and mortality.

A study by Mesnage et al. [57] on rats given an ultra-low dose of Roundup over two years found that “Overall, metabolome and proteome disturbances showed a substantial overlap with biomarkers of non-alcoholic fatty liver disease and its progression to steatohepatosis and thus confirm liver functional dysfunction resulting from chronic ultra-low dose GBH glyphosate-based herbicide] exposure”.

A significant dose-dependent cytotoxicity of both glyphosate and Roundup Bioflow on in vitro human and murine cell lines (Caco2 and L929) was found by Truzzi et al. [58].

As part of its comprehensive study of glyphosate, the Ramazzini Institute has published some results from its pilot study. In these papers, rats were given glyphosate for 13 weeks at human-equivalent doses (the U.S. acceptable daily intake is 1.75 mg/kg bw/day) and were monitored from a prenatal stage to adulthood. Manservisi et al. [59] found effects on the endocrine system and on reproductive development. Mao et al. [60] found changes in the gut microbiome of F1 pups: *Bacteriodetes* (*Prevotella* increased while *Firmicutes* (*Lactobacillus*) decreased.

Since the introduction of genetically engineered crops in 1996 that tolerated spraying with Roundup, the use of Roundup has risen dramatically—and so has the incidence of illnesses in the United States. Strong correlations over time were found by Swanson et al. [2] between the number of deaths of Americans from various chronic illnesses in any year and the amount of glyphosate applied in that year. The diseases studied in this paper included obesity, stroke, hypertension, senile dementia, Alzheimer’s disease, Parkinson’s disease, autism, and several kinds of cancer, among the 22 diseases included in total. Most of the glyphosate, applied in the form of Roundup, was applied to maize (corn) and soya beans, both of which are widely planted.

The Detox Project [47] sampled 83 essential foods in the United States and found that more than half contained residues of glyphosate. Contaminated foods included bread (especially whole-wheat bread), chickpeas, and other pulses and grains including oats. None of the foods was genetically engineered (GM). Even some foods labelled as Non-GMO (non-GM organism) had glyphosate residues. Spraying before harvest to desiccate the crops is mainly responsible for the residues. From 1993 (before any GM crop was marketed) to 2016, there was an increase by as much as 1208% in the levels of glyphosate in the urine of people in Southern California who were tracked during this time. As would be expected, organic foods had the lowest levels of glyphosate.

A study by Grau et al. [61] found glyphosate in the urine of 99.8% of the 6795 samples taken among the general population of France at various seasons.

Hao et al. [4] investigated the effects of glyphosate alone and also of glyphosate together with ethoxylated formulants and their mixtures on cell lines of the human liver, lungs and nerve tissue. The formulations were found to be toxic, inhibiting cell proliferation, while glyphosate alone had no significant effect. However, there are also studies showing that glyphosate itself is toxic.

## 6. Discussion

As people everywhere gradually become aware of the dangers posed by glyphosate-based herbicides, they are demanding that action be taken to terminate their use.

A Citizens’ Initiative in 2016 was a petition for the EU to “Ban glyphosate and protect people and the environment from toxic pesticides” [62]. It attracted over one million signatures, thus triggering consideration by the European Commission and possibly bringing about a new law [63]. On 27 March 2021, this initiative did indeed bring into law a new Transparency Regulation “with the aim of increasing the transparency of risk assessment in the food chain, to strengthen the reliability, objectivity and independence of the studies submitted to EFSA, and reinforce the governance of EFSA” [64].

The group Secrets Toxiques [65] has a campaign and a petition to withdraw several toxic products including glyphosate from the EU market.

Increasingly, regions and states in the United States and in countries around the world are banning or considering bans on glyphosate-based herbicides; for example, Austria banned glyphosate in 2019 [66] and Germany is planning a ban from 2024 [67].

Short of a ban on glyphosate-based herbicides, they should at least be assessed by regulators. However, this is not always carried out, and only ‘active principles’ are assessed.

In the United States, many people have suffered serious health impairment or death from non-Hodgkin’s lymphoma, which they claim to have been caused by exposure to Roundup. One of the findings of the IARC report on glyphosate [31] (see Section 5.2 above) was that a “positive association has been observed for non-Hodgkin lymphoma”. By 2021, 125,000 such cases had been brought to court against Monsanto/Bayer [68]. During the first trial (which found in favour of the plaintiff [69], with huge financial compensation), a large number of Monsanto’s private internal documents including emails was deposited with the court. When Monsanto failed to respond to warnings that it had to request confidentiality for these documents within a time limit, the court was free to make them public. The resulting ‘Monsanto Papers’ were published online [39,70] and revealed the covert manipulations by Monsanto to destroy the credibility of studies by independent scientists revealing the harm caused by the use of Roundup.

If glyphosate were banned, GM crops with a single GM trait to tolerate Roundup would have no purpose, and GM crops that incorporate Roundup tolerance, along with other traits, would be less valuable. The result would be a crippling loss of profits for companies selling such seeds; this is the reason for the tenacious insistence of pesticide- and GM-companies that glyphosate and its formulations are safe.

A heated debate continues over the safety of glyphosate and Roundup. The small-scale study by Séralini et al. [40,43] found serious damage to the health of rats over their lifetime from the consumption of Roundup at real-life levels in drinking water (see Section 5.2). To provide a definitive scientific resolution as to the safety of glyphosate, the Ramazzini Institute has undertaken the most comprehensive study of the effects of glyphosate-based herbicides at real-life levels [71]. Subjects for the study will include carcinogenicity, long-term toxicity, neurotoxicity, endocrine disrupting effects, prenatal developmental toxicity, the microbiome, and multi-generational effects. To remain independent, it receives no financial support from the industry and depends on worldwide crowdfunding.

## 7. Conclusions

The regulation of glyphosate in the European Union and in the United States is corrupted by malpractices arising from conflicts of interest. Officials from the industries that produce pesticides and genetically modified crops are placed in high positions in the regulatory agencies, and the actions of the agencies are then steered towards ensuring a favourable outcome for applications to bring to market the products of those industries.

Sweeping regulatory reform is urgently needed to prevent further harm to humans, animals, and the environment. In spite of the large and growing number of papers published by independent scientists revealing the harm, the assessment processes have largely dismissed these papers on bureaucratic technicalities. The long-term toxicity of the formulations needs to be included but is not part of the regulation by the EFSA, ECHA and EPA, and was not part of the work of IARC. Governments and regulators need to understand that their primary purpose is to promote the welfare of the population and the environment. At present, governments are narrowly focused on bolstering the economy via the huge profits made by giant corporations selling pesticides and GMOs, and regulators work closely with these industries. The result of these practices is that the health of people and animals suffers, and the environment becomes degraded.

It is time to excise the corruption.

## Data Availability

Not applicable.
